# From the Editor's Desk

**Published:** 2017

**Authors:** Patrick Commerford

## From the Editor's Desk

The peer-review process is a vital part of scientific publication and seeks to ensure that what is published has been effectively scrutinised for scientific integrity, validity and ethical conduct in research. No matter how good peer review may be it is inevitably, by its very nature, limited to the opinions of a small number of reviewers and editors.

An important part of peer review, little mentioned, occurs after publication when the published work is exposed to a very much wider audience. This readership is often in a better position to offer critical opinions or commendations than the initial reviewers and is able to make its opinion known through letters to the editor. When these are published they offer important insights into the merit or otherwise of prior articles and serve an important educational purpose.

Sadly of late there have been few such letters submitted to this journal. I encourage all readers of this journal to consider submitting letters of criticism or acclaim to the journal for consideration for publication. Ideally they should be brief and to the point, referencing the article under discussion with no more than three to five additional references. All letters will be submitted to the author of the original article, offering a right of reply. The letter and response, if forthcoming, will be published together. Letters to the editor (and the response) will not be subjected to further review but will be accepted or rejected based on the opinion of the editor.

The contributions by readers criticising or commenting on published work are an important part of scientific and clinical responsibility for all of us and I encourage all readers to participate actively so as to enhance the scientific integrity and value of the CVJA. Detailed instructions for authors of letters to the editor will be added to the website shortly but in the interim I will gladly accept submissions under the conditions outlined above.

In an attempt to diversify the content of the journal and to cater for the ever-growing importance of diverse imaging modalities, I plan to develop a series of ‘Images in Cardiology’. The exact requirements will be posted in the instructions for authors on the journal website shortly. In the interim, I invite the submission of suitable images for consideration for publication. The images should be of high quality and suitable for publication, as already specified on the website. They should be accompanied by a brief clinical vignette, a report of why the imaging modality was chosen and how it contributed to patient outcome. A description of the results of imaging, suitably labelled with arrows or other markers, indicating areas of particular interest is essential. A maximum of five references may be supplied. Priority will be given to images of cardiac diseases commonly seen in Africa. Submissions will be subject to peer review by experts in the field.

With the dramatic developments that have occurred in the field of imaging and that have revolutionised cardiovascular diagnosis over the last several decades, some ‘old standards’ seem to have fallen away and receive less attention than they did previously. One such is electrocardiography, which many believe, as I do, still serves as an essential aid to clinical diagnosis. It is cheap, reproducible, non-invasive and readily available. I hope to develop a series of ECG presentations to be produced on a regular basis. Other cardiovascular journals publish an ‘ECG quiz’ and I enjoy them. I sense however that some such quizzes are often difficult for the average clinician, such as myself, and therefore are avoided. My plan is to provide educational ECGs linked to clinical cases, which would be of interest to physicians and clinical cardiologists, rather than specialist electrophysiologists.

I hope that these new initiatives will meet with your support and that you will continue to contribute to the journal and criticise or comment in letters to the editor when you find it necessary. Please feel free to contact me, by a letter for publication, as above, or via the journal e-mail, with suggestions as to how the journal could be improved to better meet your needs.

